# A novel hydrazide compound exerts anti-metastatic effect against breast cancer

**DOI:** 10.1186/s40659-019-0247-2

**Published:** 2019-08-06

**Authors:** Soudeh Dehghani, Zahra Kooshafar, Ali Almasirad, Raheleh Tahmasvand, Fariborz Moayer, Ahad Muhammadnejad, Samira Shafiee, Mona Salimi

**Affiliations:** 1 Department of Physiology and Pharmacology, Pasteur Institute of Iran, Tehran, Iran; 2 Department of Medicinal Chemistry, Faculty of Pharmacy, Tehran Medical Sciences, Islamic Azad University, Tehran, Iran; 3 Department of Pathobiology, College of Veterinary Medicine, Karaj Branch, Islamic Azad University, Alborz, Iran; 4 Cancer Biology Research Center, Cancer Institute of Iran, Tehran University of Medical Sciences, Tehran, Iran

**Keywords:** Metastasis, Breast cancer, Matrigel, Immunohistochemistry, Soft agar

## Abstract

**Background:**

There are currently a number of barriers hindering the successful treatment of breast cancer, including the metastatic spread of cancer cells. In looking for new anticancer agents, we reported two novel hydrazide derivatives with anti-cancer activity in human breast cancer cells. The current study aims to explore the therapeutic potential of the most effective one, N'-((5-nitrothiophen-2-yl)methylene)-2-(phenylthio)benzohydrazide (compound **B**), on metastatic breast cancer, which is resistant to available chemotherapeutics.

**Methods:**

4T1 mammary carcinoma cells were inoculated into the fat pad mammary of 5–7-week-old female BALB/c mice and then the effective compound was intraperitoneally administered for 4 weeks. Proliferation index and angiogenesis in tumor and lung tissues were examined with immunohistochemistry. In vitro assessments were also carried out to evaluate the effect of the compound on invasion of MDA-MB-231 cells.

**Results:**

Our results demonstrated that this effective derivative significantly inhibited invasion of MDA-MB-231 cells in vitro as shown by Matrigel assay and quantitative real-time method for MMP-9 expression after 48 h of treatment. Daily administration of the compound suppressed the growth of primary tumor and its metastasis to lung, which was confirmed by H&E experiment at a dose of 1 mg/kg in a well-known metastatic model of 4T1 breast cancer in syngeneic BALB/c mice. These outcomes were supported by the immunohistochemical examinations of the tumor and lung tissues of mice. Tumors and lungs in mice treated with the effective compound showed a reduced proliferation index and a smaller microvessel density compared to the control.

**Conclusion:**

This study highlights an anti-metastatic role for a novel hydrazide derivative in both in vitro and in vivo models of breast cancer.

**Electronic supplementary material:**

The online version of this article (10.1186/s40659-019-0247-2) contains supplementary material, which is available to authorized users.

## Background

Cancer remains as the most common devastating health problem throughout the world, with an increasing incidence and mortality rate [1]. Among different types of cancer, breast cancer is regarded as a dominant cause of cancer-associated mortality in women with an upward trend in incidence in the developing countries including Iran [2]. Based on a 2015 statistical report in Iran, 6160 new breast cancer cases were diagnosed of which 1063 cases led to death [3]. Mortality of breast cancer is normally due to tumor metastasis, defined by a high potential of spreading to other sites [4]. Metastasis is a multistep biological process involving cell migration, invasion and angiogenesis [5, 6]. Discovering novel agents with the ability to suppress more than one step would be promising in drug discovery. Triple-negative breast cancer (TNBC) is associated with high invasiveness and poor prognosis, which frequently relapses following treatment [7, 8]. Lack of a proper targeted therapy along with a resistance to chemotherapy has made this subtype of breast cancer a major concern [9]. To overcome this issue, mouse mammary cancer 4T1 cell line has been used in establishing an animal model with a high metastatic feature in BALB/c mice, recapitulating breast cancer in human body [10]. Using this murine model has become a fundamental tool for the development of chemotherapeutic agents with improved clinical trial outcomes [11].

Treatment approaches for metastatic breast cancer have been gradually developed in recent years; however, chemotherapy remains a major strategy. Several limitations have been reported of chemotherapy, including tumor drug resistance and risk of toxicity [12]. Thus, designing novel compounds possessing potent as well as selective cytotoxic property is undoubtedly of great importance. In this regard, compounds containing hydrazone as a fragment were found to display a wide range of biological activities including anticancer effect [13]. There are also reports on a hydrazide–hydrazone moiety in synthetic compounds having a decisive role in anticancer activity, however with little information on their anti-metastatic property [14].

**Fig. 1:**
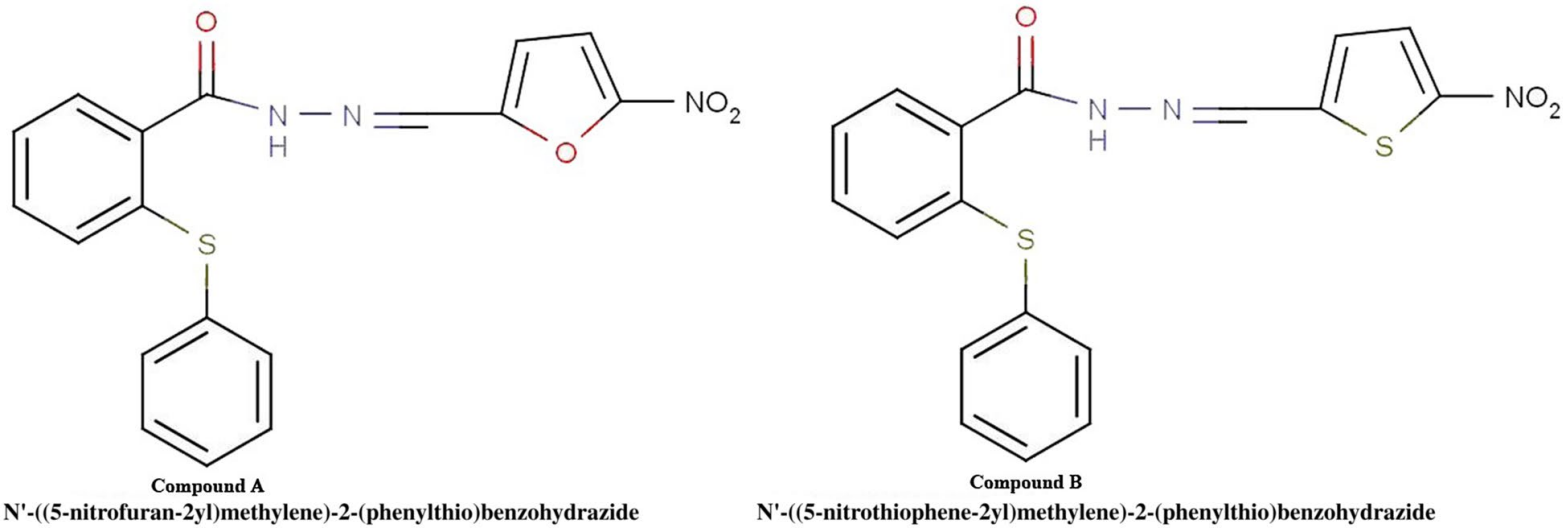
Chemical structures of compounds **A** and **B**

Considering the aforementioned reports demonstrating the potential anticancer activity of compounds **A** and **B** (Fig. 1) [15], we selected the most effective compound (compound **B**) to further investigate its anti-metastatic property in an in vivo model system. To the best our knowledge, this is the first report showing the anti-metastatic activity of this compound.

## Methods

### Cells and chemicals

Human breast adenocarcinoma (MDA-MB-231, C578) and mouse mammary tumor (4T1, C604) cell lines were obtained from NCBI. DMEM and FBS were prepared from GibcoBRL (Rockville, IN, USA). Matrigel basement membrane matrix was purchased from BD Biosciences (San Jose, USA). Compound **B** was synthesized in the medicinal chemistry research laboratory in the Faculty of Pharmacy, Tehran Medical Sciences, Islamic Azad University [15, 16]. All chemicals used, if not indicated, were purchased from Merck (Darmstadt, Germany) and Sigma-Aldrich (St Louis, MO, USA).

### Matrigel-based invasion assay

To reveal the effects of compound **B** on the invasive behavior of MDA-MB-231 cells in vitro, changes in invasiveness of cancer cells were assessed. To measure cell invasion, migration of cancer cells from a porous membrane was tested in a Transwell 24-well polycarbonate permeable support with a 8 µm pore diameter (SPL Life Sciences, Korea). The porous membrane in each well was coated with 45 µl of matrigel, an ECM gel, with a final concentration of 1 mg/ml in serum-free DMEM on ice.

MDA-MB-231 cells were treated with compound **B** (0.4 µM equivalent to 2 × IC_50–72h_ concentration) for 48 h and serum-starved overnight [15]. Following cell re-suspension, they were seeded into the upper wells (inserts) of the invasion chamber with serum free medium at a concentration of 1 × 10^5^ cells/well. Following removal of the remaining cells in the inserts after 24 h by a cotton swab, cells on the underside of the membrane were washed with PBS. Cells were then fixed using 4% formaldehyde, stained with 0.5 mg/ml crystal violet and observed under an inverted microscope.

### Soft agar colony assay

MDA-MB-231 cells treated with compound **B** and MDA-MB-231 cells alone were suspended in 0.35% agarose and 2X DMEM supplemented with 20% FBS and seeded over a basal layer of 0.5% agarose. The experiments were established in 100 mm petri dishes at a cell density of 6 × 10^3^ cells/well in triplicates. Upon staining with crystal violet, colonies were manually counted in nine random fields after 21 days of culture at 37 °C. Phase contrast micrographs of the colonies were captured with a Nikon microscope (Nikon, Tokyo, Japan).

### RT-qPCR analysis of mRNA expression

Total RNA was isolated by Trizol reagent (Invitrogen, USA). The cDNA synthesis kit (ThermoFisher Scientific, USA) was used following the manufacturer’s instruction. The relative mRNA expression data was analyzed using the 2^−ΔΔCt^ method with GAPDH RNA used as a reference gene. Primers were designed by AlleleID6 software (PREMIER Biosoft International, USA). Gene sequences of MMP-9 and GAPDH were obtained from GenBank (NCBI, BethesdaMD, USA) (NM_004994.3 and NM_002046.5), and BLAST analysis (NCBI) was performed on the primer pair to evaluate their specificity.

### Animals

Eighty female BALB/c mice (5–7 weeks, 18–20 g weights) were provided by the National Animal Center (Pasteur Institute of Karaj) and maintained in a 12/12-h light–dark cycle, with food and water supplied *ad libitum*. Animals were treated in keeping with the guideline approved by the animal ethics committee of Pasteur Institute of Iran.

### Experimental tumor model and treatments

Exponentially 4T1 cells were trypsinized, suspended in PBS, and 10^6^ cells were inoculated into the mammary fat pad of the mice to obtain a solid tumor growth. Seven days after inoculation, once the tumor masses were palpable, the mice were assigned in 8 groups of 10 animals each, as follows: Animals were treated with intraperitoneal injections of 1, 10 and 50 mg/kg of compound **B** five days a week for 4 weeks. The control vehicle group received 20 µl of DMSO. Tumor sizes were periodically measured every 3 days. The volume of the tumor was determined using the following equation: tumor volume (mm^3^) = (length × width^2^)/2, where the length and width are in mm [15]. Following the sacrifice of the animals, the tumors and lungs were dissected from the animals for histopathological and immunohistochemical analysis.

### Histopathology

Tumor and lung tissues were fixed in formalin (10%) and then parafinized, trimmed and sliced into 5 µm slices. Following mounting the slice on a glass slide, tissue slides were deparaffinized, rehydrated and subjected to H&E staining. Selected fields were randomly photographed at 400× magnification using a Carl Zeiss AxioImager microscope and Image M1 Software (Carl Zeiss, Jena, Germany).

### IHC staining

To assess proliferative cell percentage by Ki-67 staining, paraffin-embedded tumor sections (4 µm) were deparaffinized in xylene and rehydrated by graded alcohol and then incubated with anti-Ki-67 antibody (ab15580, Abcam; dilution 1:200) followed by biotinylated secondary antibody using an HRP/DAB detection IHC kit (ab64264, Abcam, Cambridge, MA 02139-1517, UK) according to the manufacturer’s instructions and finally analyzed by an expert pathologist. Positive cells were stained brown and accounted for proliferating cells expressing Ki-67. Cells stained blue by hematoxylin were considered as negative cells. The percentage of positive tumor cells within one high-power field was accounted for IHC expression level of Ki-67, and classified into four grades (negative, < 1%; low, 1–10%; moderate, 10–50%; high, > 51%) [17, 18]. To evaluate CD31 expression, tumor sections were immunostained with an anti-CD31 antibody (ab28364, Abcam, 1:50 dilution) to observe blood vessels. Brown-stained vessels were counted in selected fields with 400× magnification, and the mean microvessel density was recorded.

### Statistical analysis

The results were shown as mean ± SEM of at least triplicates, and data were compared based on one-way ANOVA followed by the Tukey’s post test using GraphPad Prism 6.0 Software. *p* < 0.05 was considered as significant.

## Results

### Anti-invasive properties of compound B

**Fig. 2:**
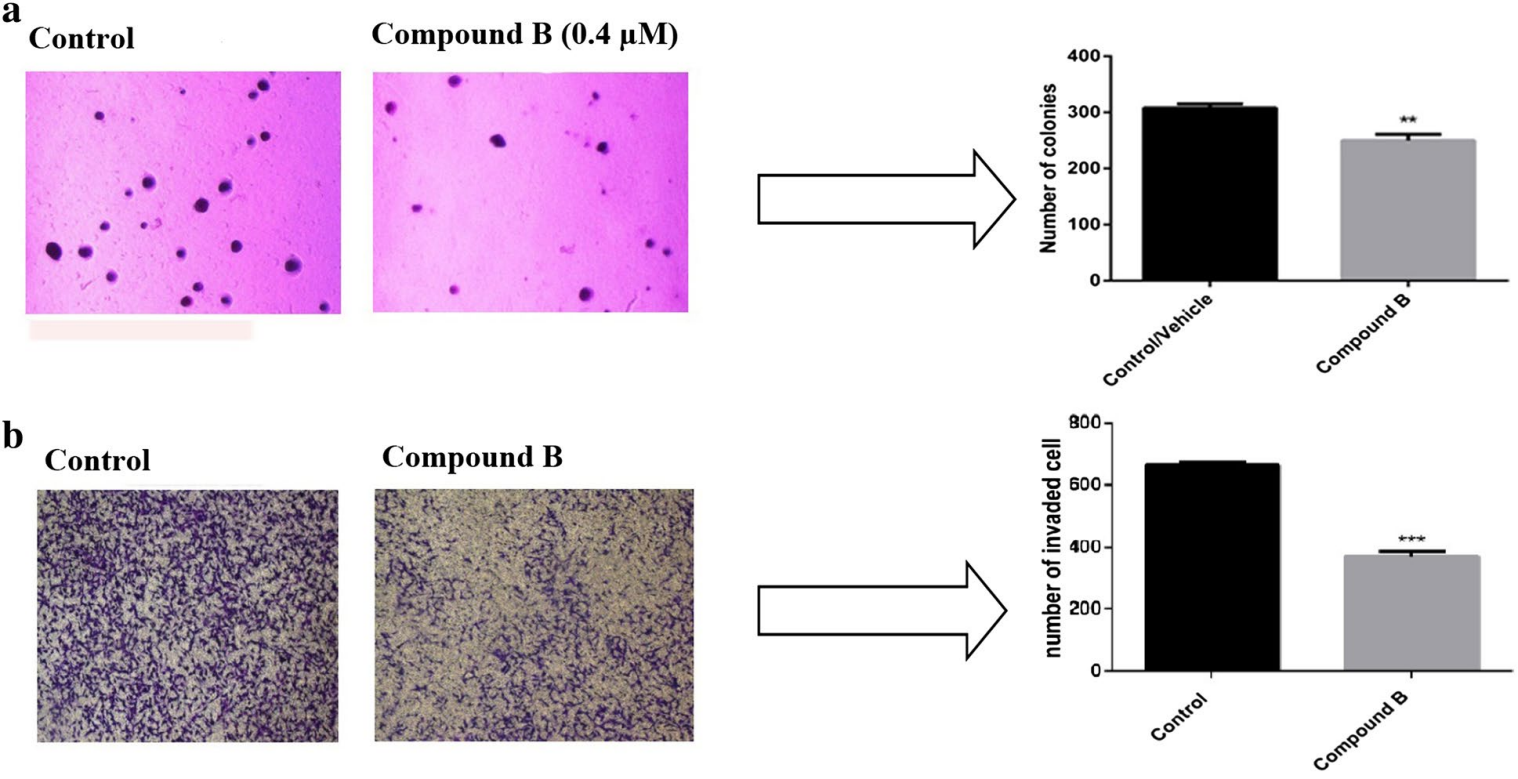
Effect of compound **B** on anchorage independent growth and invasiveness of cancer cells using soft agar colony formation assay (**a**) and matrigel-based invasion of MDA-MB-231 cells for 24 h (**b**). Number of colonies was counted in five randomly selected fields in each well under an inverted microscope (×400). Error bars represent three independent samples in triplicate repeats, and data are presented as mean ± SEM, one-way ANOVA analysis with Tukey post test was performed (**p < 0.01, ***p < 0.001 comparing to the MDA-MB-231 cells)

In order to determine anchorage-independent growth and self-renewal of breast cancer cells in the presence of compound **B**, we applied the soft agar colony formation assay and defined as the ability of the cells to independently grow on a solid surface [19]. As shown in Fig. 2a, MDA-MB-231 cells treated with compound **B** at 0.4 µM showed fewer colonies than untreated MDA-MB-231 cells representing the ability of compound **B** to effectively repress the anchorage-independent growth of the breast cancer cells. The findings from matrigel experiment indicated a reduced invasive property of MDA-MB-231 cells treated with compound **B** at 0.4 µM as evidenced by the decreased number of invaded cells (Fig. 2b). We additionally evaluated compound **A** at 1.4 µM. To achieve this, we treated MDA-MB-231 cells with 1.4 µM of compound **A** and observed that this compound caused less consequence on the invaded cells and also less number of colonies in soft agar formation compared with MDA-MB-231 cells alone after 48 h (Additional file 1: Fig. S1).

**Fig. 3:**
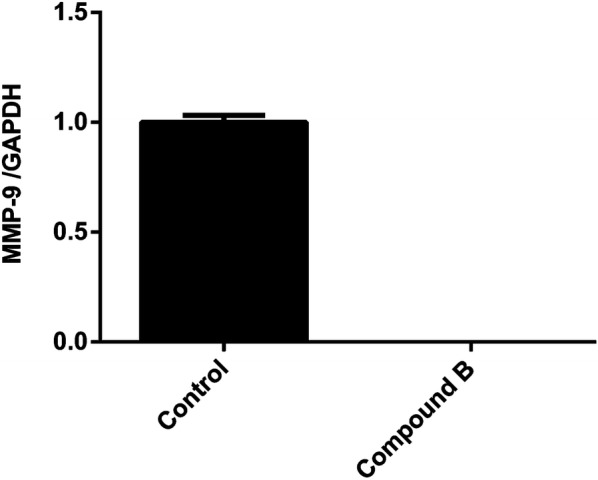
MDA-MB-231 cells were incubated with compound **B** (0.4 µM) for 48 h. The cells were subsequently assayed for MMP‑9 mRNA expression by semiquantitative RT‑PCR. Results are presented as the mean ± SEM of three independent experiments

MMP-9 as an essential marker for tumor vasculogenesis and metastatic potential of cancer was highly expressed in the invasive types of breast cancer [20, 21]. MMP-9 activity was modulated at three stages: gene transcription, post-transcriptional activation of zymogens, and endogenous expression of tissue inhibitor of metalloproteinases [22, 23]. The present study revealed that compound **B** strikingly diminished the expression of MMP-9 at the transcriptional level at concentration of 0.4 µM (Fig. 3). However, a reduction in mRNA expression level following treatment with compound **A** at 1.4 µM was visualized (Additional file 1: Fig. S2). MDA-MB-231 cells displayed a basal level of MMP-9 mRNA expression.

### Tumor growth suppression by compound B in BALB/c mice

**Fig. 4:**
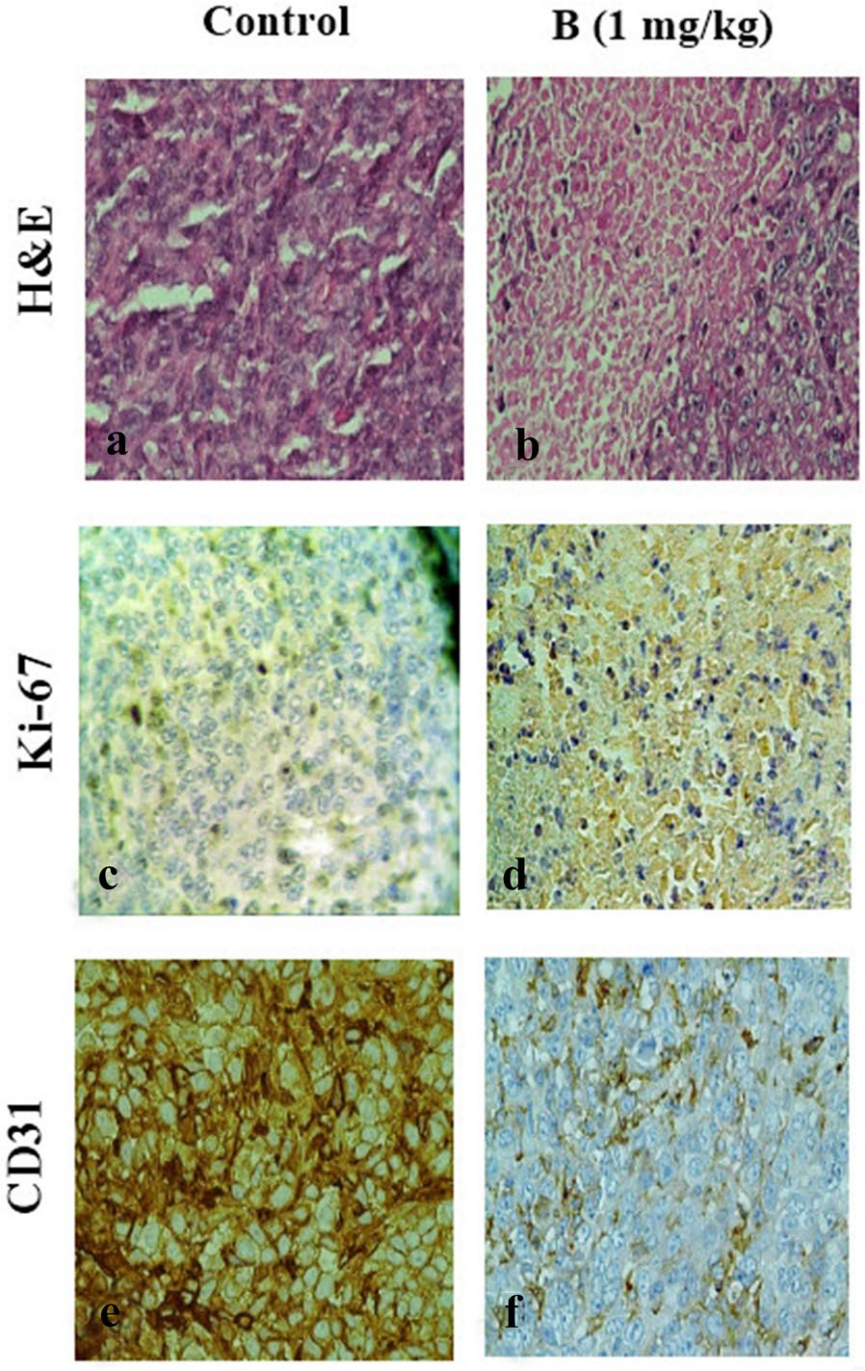
Effect of compound **B** on solid tumors in BALB/c mice injected with 4T1 cells. The mice were killed 32 days after cell injection, and tumor sections were evaluated by H&E staining (**a**, **b**) and immunostaining detection of Ki-67 (**c**, **d**) and CD 31 (**e**, **f**) (original magnification ×400)

The volume of a large 4T1 tumor detected in the control group was reduced the mice treated with compound **B** (Additional file 1: Fig. S3) [15]. Morphological changes in cells, including loss of polarity due to pleomorphism and a large amount of typical and atypical mitosis, were obvious in tumors dissected from the 4T1 administered mice, indicating the high malignancy of 4T1 cells (Fig. 4a). These types of cells, designated as an invasive lineage, represent an appropriate model for assessing the efficacy of anticancer drugs due to its similarities with metastatic human breast cancer cells. Following treatment with compound **B**, a great number of tumor cells were in the process of necrosis, and areas of hemorrhage could be detected in tumor tissues (Fig. 4b). In addition, we performed this experiment on the mice treated with compound **A** (Data shown in Additional file 1: Fig. S4). These results corroborate the findings of our previous study demonstrating a high potency for compound **B**.

In line with our histopathological data, IHC staining revealed a considerable reduction in the proportion of Ki-67 (prototypic cell cycle related nuclear antigen) and CD31 (platelet/endothelial cell adhesion molecule; PECAM-1) in the mammalian tumors from compound **B**-treated group compared to the vehicle-administered mice (Fig. 4c–f). As shown in Fig. 4d, Ki-67 count in 1 mg/kg/day compound **B**-treated group was 6.5 ± 1.5%, while this index was 44.3 ± 3.3% in the control group mice (Fig. 4c), confirming a high effectiveness of compound **B**. Moreover, the tumor sections were stained with an anti-CD31 antibody to report microvessel density (Fig. 4 e, f). Mean MVD in the invading tumor areas was 35 vessels, whereas this value was 25 for compound **B** (1 mg/kg/day) treated group (data for compound **A** was shown in Additional file 4: Fig. S4). Based on the results, 1 mg/kg of compound **B** treatment led to a great reduction in tumor-induced neovascularization.

### Compound B diminished metastatic progression of 4T1 cells to lungs in the treated mice

**Fig. 5:**
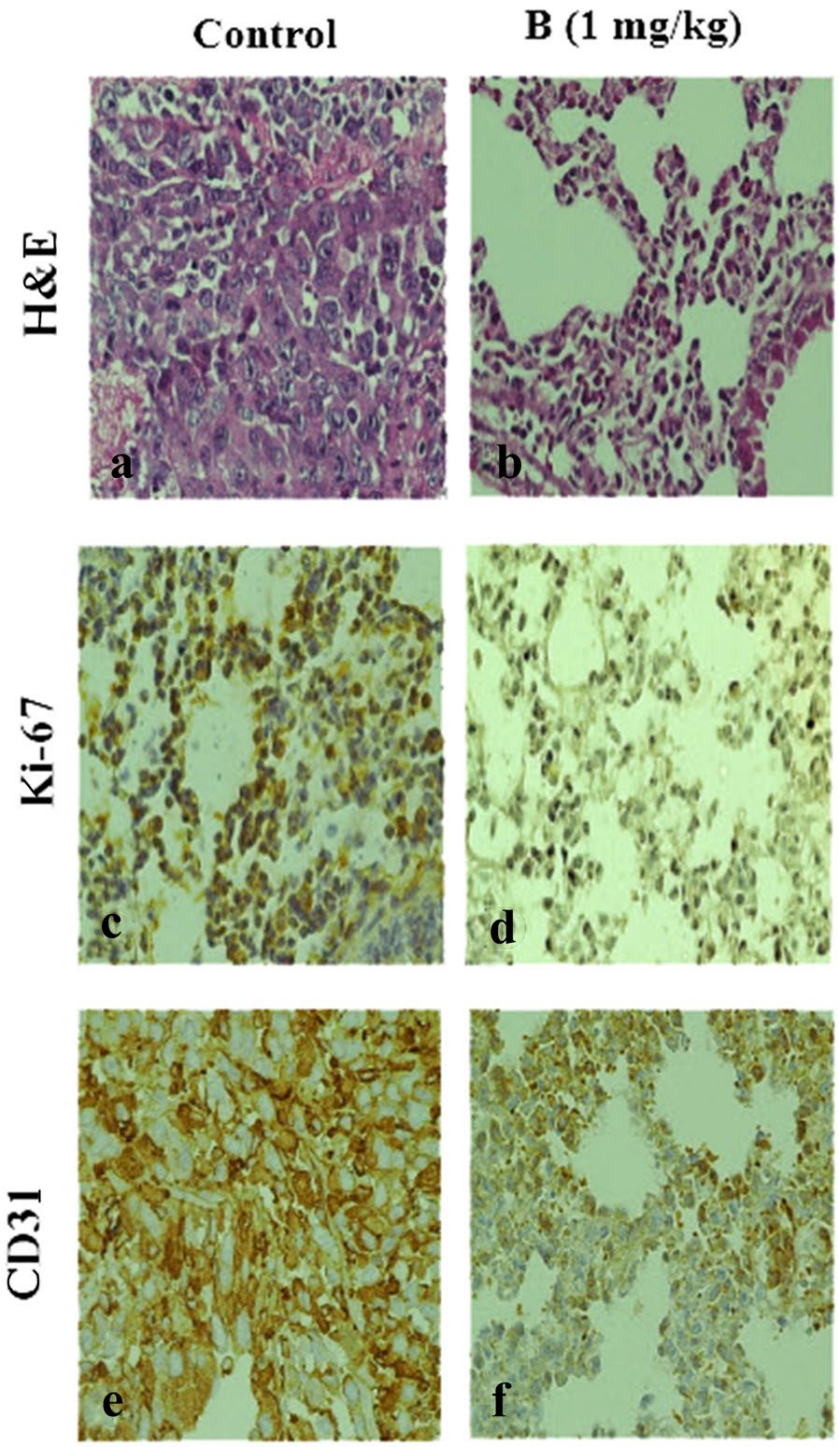
Compound **B** inhibits metastasis to the lungs of BALB/c mice inoculated with 4T1 cells. H&E staining was performed on the lungs (**a**, **b**). Upon treatment, lungs were sampled to evaluate the extent at which the cells had metastasized. Lung sections were stained with antibody raised against Ki-67 (**c**, **d**) and CD31 (**e**, **f**), and then with 1,3-diaminobenzidine (DAB) and counterstained with hematoxylin. Representative images of the immunohistochemical analysis are shown. All photomicrographs are at ×400 magnification

Lung metastasis was evaluated in order to reveal the therapeutic potential of the compound in inhibiting the outgrowth of disseminated metastatic tumors. A marked reduction (Fig. 5) in breast cancer metastasis to the lungs was observed upon treatment with compound **B** (for compound **A** referred to Additional file 5: Fig. S5). These results displayed the suppressive effect of compound **B** (Fig. 5b), on the migration of breast cancer cells from their origin sites to the lungs as demonstrated by our in vitro migration assay. CD31 analysis further confirmed the absence of micro-metastatic lesions in the lungs of mice administered with 1 mg/kg/day of compound **B** (Fig. 5f). The in vivo and in vitro data, thus far, are suggestive of the anti-metastatic effect of compound **B** being attributable to its anti-angiogenic potency. Finally, to score the proliferating cells in lung tumors, calculations of Ki-67 positive cells were performed based on microscopic assessment of Ki-67 stained lung slices (Fig. 5 c, d). Compound **B** was potently able to diminish lung tumor proliferation (Fig. 5 b). Our results demonstrated a Ki-67 proliferation index of less than 2.5 ± 0.5% for compound **B** treated mice, compared to 50 ± 5.8% for the control group. The latter data is indicative of a lower proportion of Ki-67 brown-stained positive cells in the mice treated with compound **B**. These observations are in parallel with a lower CD31 expression detected in the secondary tumor tissues (IHC data for compound **A** were shown in Additional file 5: Fig. S5).

## Discussion

To explore the anti-metastatic effect of compound **B**, we used a 4T1-based mouse model recapitulating the multi-organ metastasis of human breast cancer. Fat pad mammary injection of 4T1 tumor cells leads to local tumor growth and subsequently metastasis to the lungs. In this study, the syngeneic mouse model was selected due to its high reproducibility and suitability for testing the efficiency of chemotherapeutic agents used in treating the metastatic breast cancer [24]. Moreover, the syngeneic mouse tumor model allows the tumor cells to metastasize effectively showing the metastatic features similar to those of breast cancer patients [25]. We have previously addressed the anti-breast tumor activity exhibited by compounds **A** and **B** [15], herein, we further extend our study to report on the potential metastasis suppressing effect of the most effective compound (compound **B**) using this model. However, for completeness, we have additionally included data for compound **A** in additional figures.

In line with our soft agar results showing the ability of compound **B** to inhibit the anchorage-independent growth of MDA-MB-231 cells and a consequent anti-tumorogenic activity, the anti-invasive effect of the compound on breast cancer cells was also confirmed by the matrigel assay. Three major steps involved in penetration of tumor cells into the basement membrane during metastasis are as follows: Displacing from the original site, entering the blood circulation, and migrating from blood flow into distant locations [26]. To provoke this, a membrane filter was covered with an artificial basement in Transwell culture plates to form a structure similar to that of a natural basement membrane. In this model, invasive tumor cells can easily penetrate the membrane by inducing chemotactics [27]. Furthermore, in the current study, the anti-invasive effect of compound **B** on breast cancer cells was verified by determining MMP-9 expression. Accumulating evidence revealed that elevated expression of different MMPs is accompanied by the progression of various types of tumors [28]. Besides, tumor angiogenesis is regulated by MMP-9 expression through modulating the bioavailability of VEGF [29]. Consistently, we observed that compound **B** significantly suppressed mRNA expression of MMP-9 in MDA-MB-231 cells. Overall, our in vitro results demonstrated that compound **B** effectively contributed to inhibition of cell invasion. These encouraging results motivated us to further validate the anti-metastatic effect of this compound towards mammary tumor cells in vivo. Thus, the mice were daily administered by 1 mg/kg of compound **B** followed by dissecting the tumor and lung tissues after a 4-weeks treatment. Our H&E findings demonstrated that the growth of 4T1 primary tumors was well controlled by compound **B** at a dose of 1 mg/kg/day. The tumor inhibitory effect of compound **B** was also evaluated by establishing the Ki-67 proliferation index. Our immunohistochemical data revealed a marked reduction in the expression of Ki‐67 in 4T1 tumors of compound **B**‐administrated animals. These findings were in consistent with the H&E results indicating a high potential benefit of compound **B** in tumor growth inhibition. The antitumor effect of compound **B** was further evidenced by CD31 analysis. Noteworthy is mentioning that CD31 is expressed on endothelial cells and employed as a marker to confirm tumor angiogenesis [30]. Interestingly, impediment to angiogenesis was detected as a mechanism contributing to the antitumor effect of compound **B**. Our results also revealed a significant reduction in the expression of CD31 in compound **B-**treated mice compared to the untreated control group. Collectively, our in vitro and in vivo data are suggestive of compound **B** being capable of inhibiting invasion by suppressing tumor-associated angiogenesis. This is a first report on the anti-invasive effect upon i.p. administration of compound **B**; however, its mechanism of action needs to be fully understood.

Once 4T1 cells are injected into BALB/c mice, they quickly proliferate and develop metastasis aggressively. For this reason, 4T1 cells have been exploited to establish a useful model in order to study the late stage of breast cancer [31, 32]. To document the anti-metastatic effect of compound **B**, lung tissues of the 4T1 injected mice as the first metastatic organ were histopathologically evaluated. Our findings highlighted that in comparison to the control, treatment with compound **B** effectively diminished pulmonary metastatic foci in the mice, an indication of the former having a greater anti-metastatic potential. In IHC analysis, protein expression of Ki-67 in cell nuclei implies proliferating cancer cells and an increased expression level of Ki-67 in the lung indicates breast cancer lung metastasis [30]. In the current study, we revealed that compound **B** lowered the number of proliferative cells, in terms of percentage, in lung tumors supporting our H&E data and highlighting the significant role of this compound in reducing metastatic breast cancer in the lungs.

Considering angiogenesis as a hallmark of metastasis and tumor development and CD31 as a marker for angiogenesis, which widely expressed on the endothelial cells [30, 33, 34], further evidence for the anti-metastatic effect of compound **B** was provided by a significant impediment to the expression in lung tissues of CD31. These data are consistent with those on Ki-67 results and collectively suggest compound **B** for the first time as potential anti-metastatic agents.

## Conclusions

It is concluded that compound **B** potently suppresses tumor growth in 4T1 tumor-bearing BALB/c mice. In addition, inhibition of angiogenesis is suggested as a mechanism through which compound **B** exerts its anti-metastatic function though possible involvement of other mechanisms can not be ignored. This is a first report on the anti-metastatic effect of compound **B** as a novel hydrazide-hydrazone derivative. We believe further investigations are a prerequisite for the establishment of this compound as an effective candidate for inhibiting tumor metastasis.

## Additional files

**Additional file 1: Fig. S1.** Effect of compound **A** on anchorage independent growth and invasiveness of cancer cells using soft agar colony formation assay (**a**) and matrigel-based invasion of MDA-MB-231 cells for 24 h (**b**). Number of colonies was counted in five randomly selected fields in each well under an inverted microscope (×400). Error bars represent three independent samples in triplicate repeats, and data are presented as mean ± SEM, one-way ANOVA analysis with Tukey post test was performed (*p < 0.05 comparing to the MDA-MB-231 cells).

**Additional file 2: Fig. S2.** MDA-MB-231 cells were incubated with compound **A** (1.4 µM) for 48 h. The cells were subsequently assayed for MMP-9 mRNA expression by semiquantitative RT-PCR. Results are presented as the mean ± SEM of three independent experiments.

**Additional file 3: Fig. S3.** Compounds **A** (10 mg/kg) and **B** (1 mg/kg) affected tumor growth after 4 weeks of daily treatment. Data are expressed as mean ± SEM, n = 10 mice per group.

**Additional file 4: Fig. S4.** Effect of compound **A** on solid tumors in BALB/c mice injected with 4T1 cells. The mice were killed 32 days after cell injection, and tumors sections were evaluated by H&E staining (**a**, **b**) and immunostaining detection of Ki-67 (**c, d**) and CD 31 (**e, f**) (original magnification × 400). Tumors from the mice administered with compound **A** showed a reduced number of proliferative cells; however, a more significant inflammatory reaction was observed around tumors in this group (**b**). Compound **A** treatment at a dose of 10 mg/kg/day also resulted in a reduction in the number of proliferative cells (~ 27%) (**d**). Mean MVD in the invading tumor areas was 35 vessels, whereas this value was 29 for compounds **A** (10 mg/kg/day) (**f**).

**Additional file 5: Fig. S5.** Compound **A** inhibits metastasis to the lungs of BALB/c mice inoculated with 4T1 cells. H&E staining was performed on the lungs (**a, b**). Upon treatment, lungs were sampled to evaluate the extent at which the cells had metastasized. Lung sections were stained with antibody raised against Ki-67 (**c, d**) and CD31 (**e, f**) and then with 1,3-diaminobenzidine (DAB) and counterstained with hematoxylin. Representative images of the immunohistochemical analysis are shown. All photomicrographs are at × 400 magnification. A slight reduction in the number of 4T1 cells in the lungs of compound **A-**treated mice was detectable. No remarkable difference in the mean of MVD in the lungs of the mice treated with 10 mg/kg/day of compound **A** compared to vehicle-control group (MVD = 27 vs. 37). Ki-67 proliferation index of less than 6.5 ± 0.5% for compound **A** treated mice, compared to 50 ± 5.8% for the control group.

## Data Availability

The datasets used during the present study are available from the corresponding author on reasonable request.

## Abbreviations

ECM: Extracellular matrix
DMEM: Dulbecco’s modification of eagle medium
DMSO: Dimethyl sufoxide
FBS: Fetal bovine serum
H&E: Hematoxylin and eosin
IHC: Immunohistochemistry
i.p.: Intraperitoneal
NCBI: National Cell Bank of Pasteur Institute of Iran
PBS: Phosphate-buffered saline
TNBC: Triple-negative breast cancer
