# Supplementary figures and images for: A novel hydrazide compound exerts anti-metastatic effect against breast cancer
Source: Biol Res. 2019 Aug 6;52:40. doi: 10.1186/s40659-019-0247-2 (PMC6683344; doi:10.1186/s40659-019-0247-2)

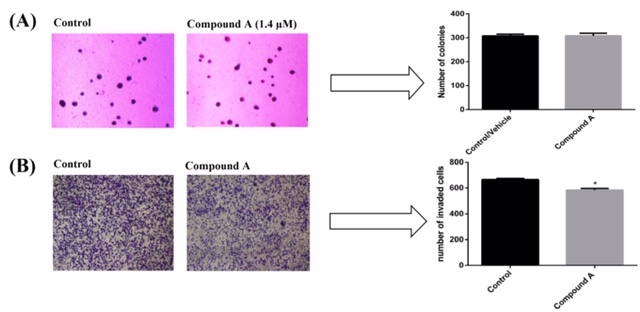

Supplement: Supplementary file 1 — Additional file 1: Fig. S1. Effect of compound A on anchorage independent growth and invasiveness of cancer cells using soft agar colony formation assay (a) and matrigel-based invasion of MDA-MB-231 cells for 24 h (b). Number of colonies was counted in five randomly selected fields in each well under an inverted microscope (×400). Error bars represent three independent samples in triplicate repeats, and data are presented as mean ± SEM, one-way ANOVA analysis with Tukey post test was performed (*p < 0.05 comparing to the MDA-MB-231 cells). [file 40659_2019_247_MOESM1_ESM.jpg]

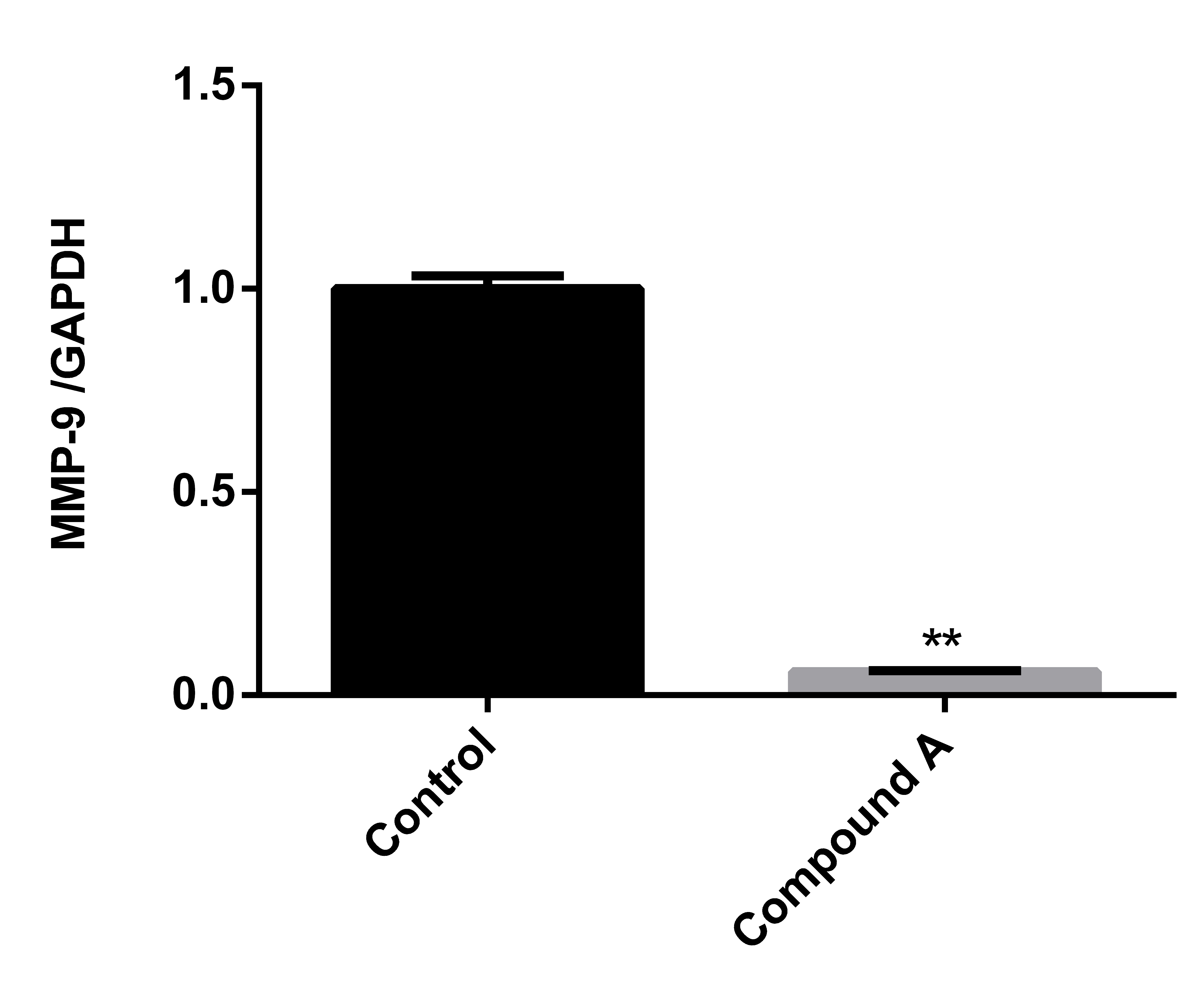

Supplement: Supplementary file 2 — Additional file 2: Fig. S2. MDA-MB-231 cells were incubated with compound A (1.4 µM) for 48 h. The cells were subsequently assayed for MMP-9 mRNA expression by semiquantitative RT-PCR. Results are presented as the mean ± SEM of three independent experiments. [file 40659_2019_247_MOESM2_ESM.jpg]

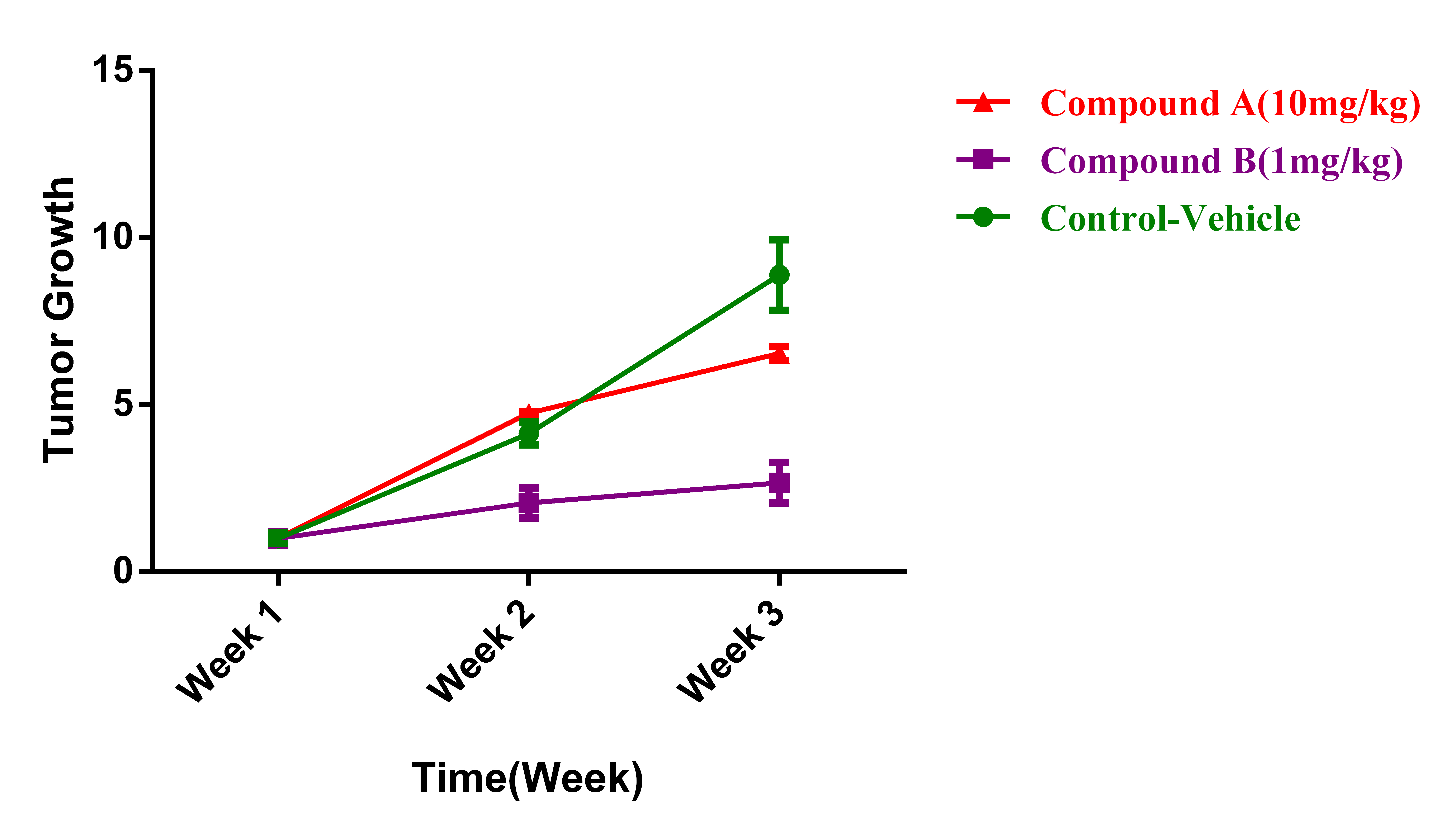

Supplement: Supplementary file 3 — Additional file 3: Fig. S3. Compounds A (10 mg/kg) and B (1 mg/kg) affected tumor growth after 4 weeks of daily treatment. Data are expressed as mean ± SEM, n = 10 mice per group. [file 40659_2019_247_MOESM3_ESM.jpg]

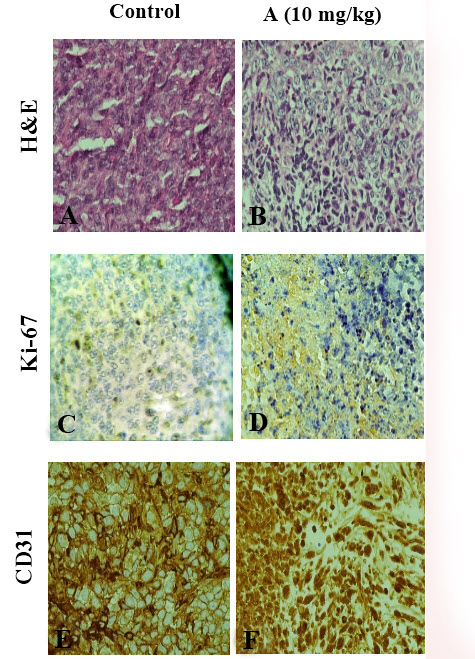

Supplement: Supplementary file 4 — Additional file 4: Fig. S4. Effect of compound A on solid tumors in BALB/c mice injected with 4T1 cells. The mice were killed 32 days after cell injection, and tumors sections were evaluated by H&E staining (a, b) and immunostaining detection of Ki-67 (c, d) and CD 31 (e, f) (original magnification × 400). Tumors from the mice administered with compound A showed a reduced number of proliferative cells; however, a more significant inflammatory reaction was observed around tumors in this group (b). Compound A treatment at a dose of 10 mg/kg/day also resulted in a reduction in the number of proliferative cells (~ 27%) (d). Mean MVD in the invading tumor areas was 35 vessels, whereas this value was 29 for compounds A (10 mg/kg/day) (f). [file 40659_2019_247_MOESM4_ESM.jpg]

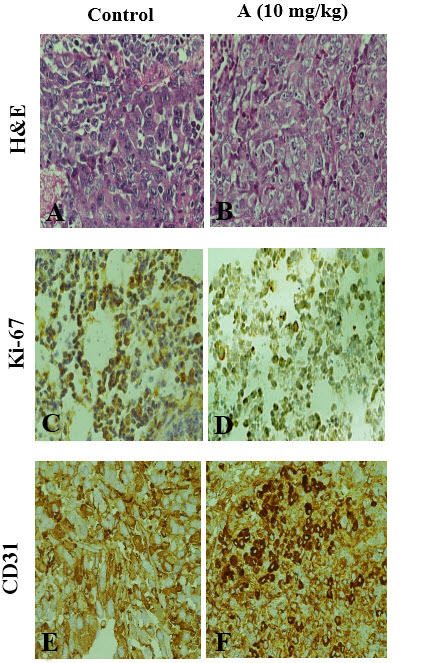

Supplement: Supplementary file 5 — Additional file 5: Fig. S5. Compound A inhibits metastasis to the lungs of BALB/c mice inoculated with 4T1 cells. H&E staining was performed on the lungs (a, b). Upon treatment, lungs were sampled to evaluate the extent at which the cells had metastasized. Lung sections were stained with antibody raised against Ki-67 (c, d) and CD31 (e, f) and then with 1,3-diaminobenzidine (DAB) and counterstained with hematoxylin. Representative images of the immunohistochemical analysis are shown. All photomicrographs are at × 400 magnification. A slight reduction in the number of 4T1 cells in the lungs of compound A-treated mice was detectable. No remarkable difference in the mean of MVD in the lungs of the mice treated with 10 mg/kg/day of compound A compared to vehicle-control group (MVD = 27 vs. 37). Ki-67 proliferation index of less than 6.5 ± 0.5% for compound A treated mice, compared to 50 ± 5.8% for the control group. [file 40659_2019_247_MOESM5_ESM.jpg]
